# Case Report: It's not always about the veins; intervention of bilateral May–Thurner Syndrome secondary to iliac aneurysm

**DOI:** 10.3389/fcvm.2023.1279981

**Published:** 2023-11-16

**Authors:** Syed H. Haq, Sidra R. Shah, Jaya Chandra, Pavithra Kannan, Sandeep M. Patel

**Affiliations:** ^1^ Department of Internal Medicine, BonSecours Mercy Health—St. Rita’s Medical Center, Lima, OH, United States; ^2^ Department of Internal Medicine, OhioHealth Riverside, Columbus, OH, United States; ^3^ Structural Heart & Intervention Center, BonSecours Mercy Health—St. Rita’s Medical Center, Lima, OH, United States

**Keywords:** May–Thurner Syndrome, aneurysm, right common iliac, thrombosis, DVT

## Abstract

May–Thurner Syndrome (MTS) remains evasive because of the insidiousness and variable etiologies by which it can manifest. In this study, we examine a unique presentation of MTS resulting from compression of both common iliac veins by a right common iliac artery aneurysm that required complex endovascular venous and arterial intervention.

## Introduction

May–Thurner Syndrome (MTS) is a condition wherein overlying arterial anatomical changes or variants may result in an unfavorable restriction of deep venous flow. This can propagate a state of venous obstruction, with subsequent development of deep venous thrombosis (DVTs) and post-thrombotic sequelae (1). In such cases, the patient may present with symptoms of acute chronic venous insufficiency, typically with the development of collaterals and thrombosis. However, a majority of cases are asymptomatic and clinically silent, thus contributing to the nebulous nature of MTS.

Traditionally, MTS is caused by a compression of the left common iliac vein (CIV) between the right common iliac artery (CIA) and the lumbar spine. However, numerous cases of CIV compression by the ipsilateral CIA have been reported (2). Regardless, thrombosis in right-sided MTS is a rare occurrence (3). Interestingly, we encountered a peculiar case of bilateral CIV obstruction and venous thrombosis resulting from a large right-sided CIA.

## Case discussion

### Presentation

A 74-year-old man presented to the emergency department with complaints of acute right-sided leg swelling, redness, and generalized discomfort. He reported that his symptoms progressively worsened over the course of 4 days and now involved the entirety of his right leg with moderate involvement of his left leg as well. He denied any recent surgical interventions, extended travels, or periods of immobility. The patient did have a history significant for a prior deep venous thrombosis and a hypercoagulable state in the setting of myelodysplastic syndrome and Factor V Leiden. For this, he was undergoing outpatient hematology and was on chronic anticoagulation. His anticoagulation was recently discontinued in preparation for an outpatient colonoscopy.

### Management

**Figure 1:**
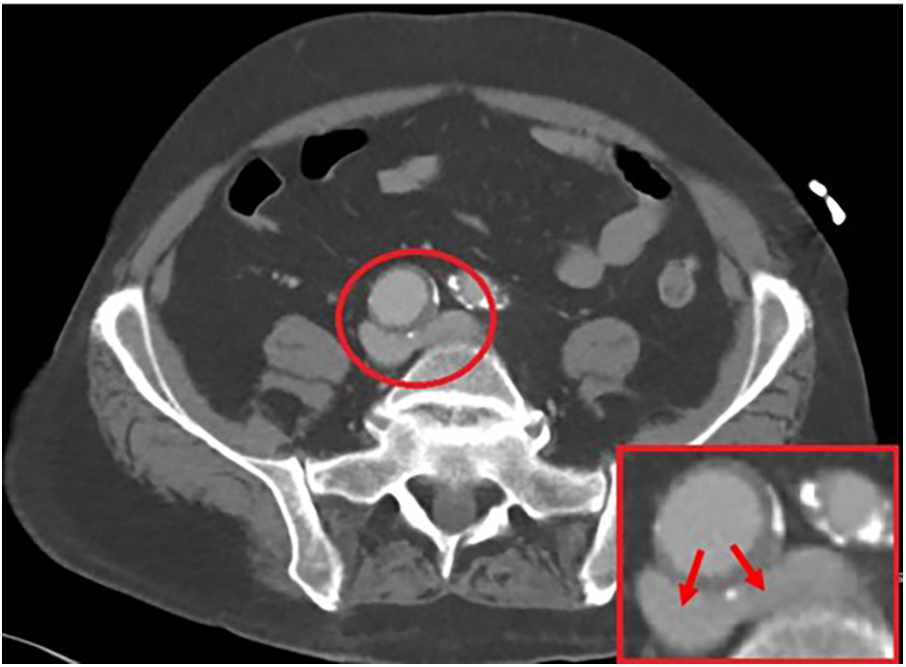
A 3.1 cm × 2.9 cm right common iliac artery aneurysm allowing for a compressive force on the bilateral iliac veins, as illustrated by the arrows.

On initial evaluation, a bilateral venous duplex study was performed, which revealed thrombosis throughout the bilateral lower superficial and deep systems. A computed tomography angiography (CTA) showed compression at the bifurcation of the iliac veins due to the presence of a large 3.1 cm × 2.9 cm right CIA aneurysm, as illustrated in Figure 1. Further investigation was sought with invasive maneuvers, as discussed in management.

**Figure 2:**
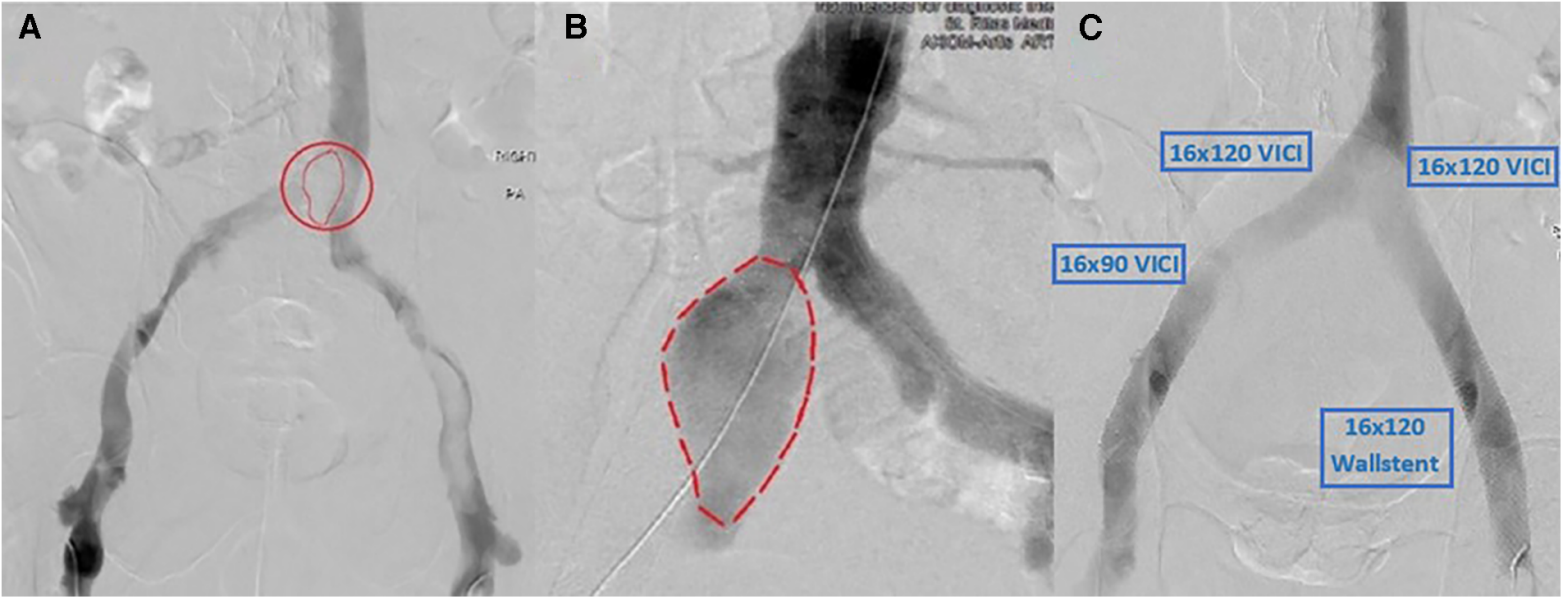
(**A**) Right CIA aneurysm causing compression of bilateral CIVs and resultant thrombosis. (**B**) Arterial angiogram showing a similar size and shape of the aneurysm that matches the lucency seen in (**A**). (**C**) Post-successful bilateral lower-extremity mechanical thrombectomy using Inari ClotTriever, followed by bilateral VICI venous stenting with an extension of the stent on the right lower extremity with a Wallstent under IVUS guidance.

The patient was taken to the catheterization laboratory where a venography revealed a significant area of overlying mobile lucency that appeared to be above the venous confluence, as illustrated in Figures 2A,B. An intravenous ultrasound (IVUS) demonstrated a pulsatile external compression of the bilateral CIV. This confirmed suspicions that an overlying CIA aneurysm was the source of hemodynamic compromise, resulting in the significant DVT burden downstream.

In the setting of two areas of concern, the acute bilateral symptomatic DVTs and the right iliac artery aneurysm, immediate intervention was deemed necessary. Vascular surgeons were consulted for possible surgery; however, the patient was not considered an operable candidate. Instead, they advised interventional cardiology evaluation for endovascular therapy. After a thorough review of the case, an interventional approach was formulated that involved addressing the thrombosed venous system with plans for staged common iliac artery intervention.

The first stage involved a prone ascending venography from the popliteal veins bilaterally with IVUS. Bilateral femoral access with a 10 French sheath was obtained. A 0.035" glide catheter was used to cross into the intravenous catheter (IVC). A standard Philips IVUS 0.035" catheter was prepped and pullbacks were performed from the IVC into both femoral veins. We then used the Inari ClotTriever (Inari Medical, Irvine, CA, USA) to perform a mechanical thrombectomy of the right and left lower extremities from the bilateral CIV to the popliteal vein. Copious amounts of acute thrombus were extracted, and post-intervention venography and IVUS confirmed adequate debulking of the thrombotic burden. IVUS was then used again bilaterally to visualize and measure stent sizing. To prevent continued compression, we elected to place bilateral kissing venous stents using IVUS guidance with bilateral 16 mm × 120 mm VICI (Boston Scientific, Marlborough, MA, USA), right-sided 16 mm × 90 mm VICI, and left-sided 16 mm × 90 mm Wallstent (Boston Scientific, Marlborough, MA, USA), as shown in Figure 2C. At the time, the VICI stent was relatively new, and given our limited experience as well as the questionable ability to use the stent across the inguinal ligament, we opted for a two-stent approach on the left with VICI at the confluence and Wallstent at the femoral head, to avoid stent fracture. There were no post-procedural complications, with a visible relief from symptoms. The patient was discharged on therapeutic anticoagulation, compression stockings, and close outpatient follow-up.

**Figure 3:**
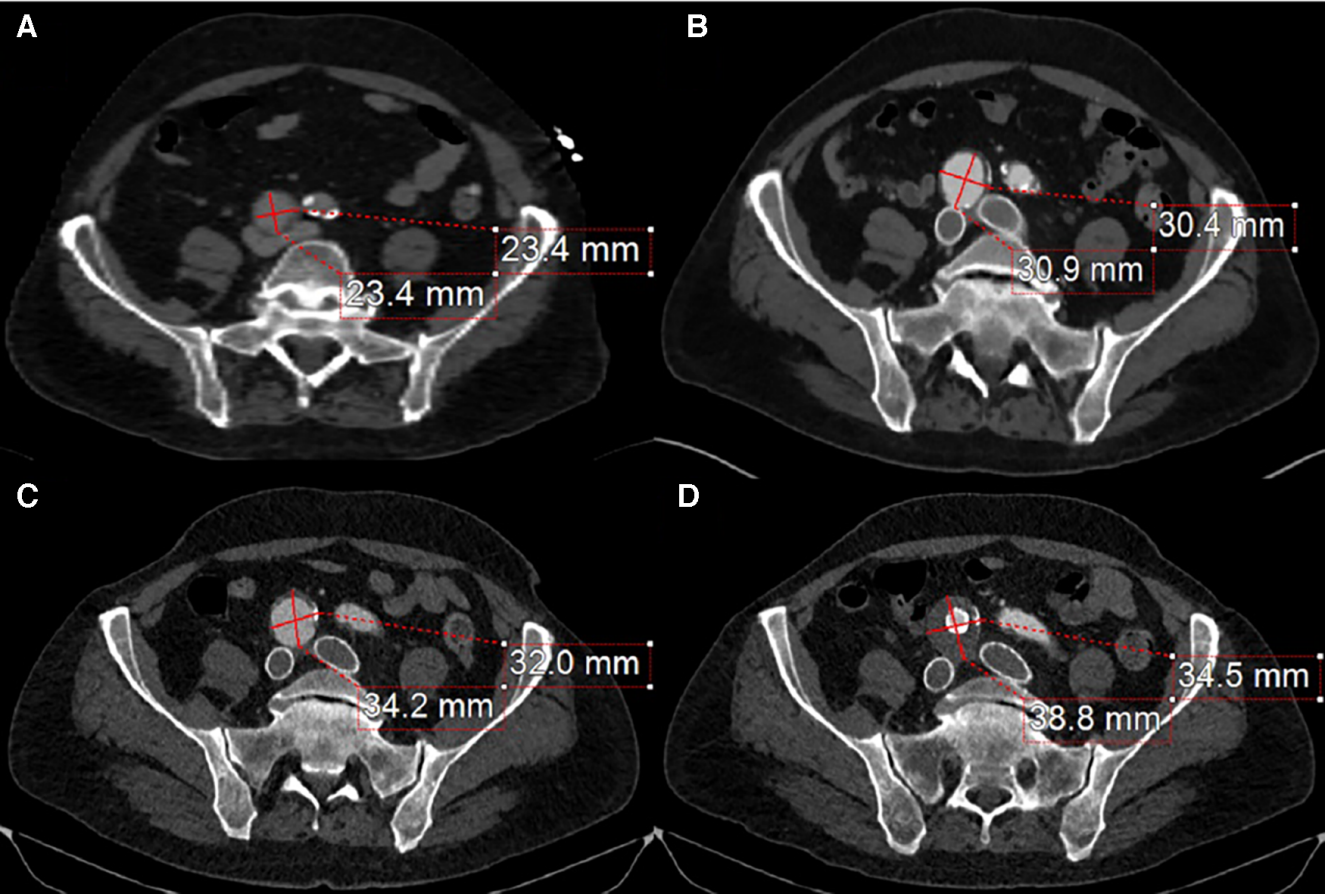
(**A**) The compressive effects of the right CIA on the bilateral iliac veins. (**B**) 4-month post-bilateral iliac venous stenting showed venous stent patency but also demonstrated an interval increase in right CIA aneurysm size. (**C**) 12-month follow-up from initial venous intervention demonstrates a progression in aneurysmal size. (**D**) 1-month post-stenting to the right common iliac artery stent graft traversing the aneurysm with a noticeable thrombus of the aneurysm sac and patent iliac artery stent.

**Figure 4:**
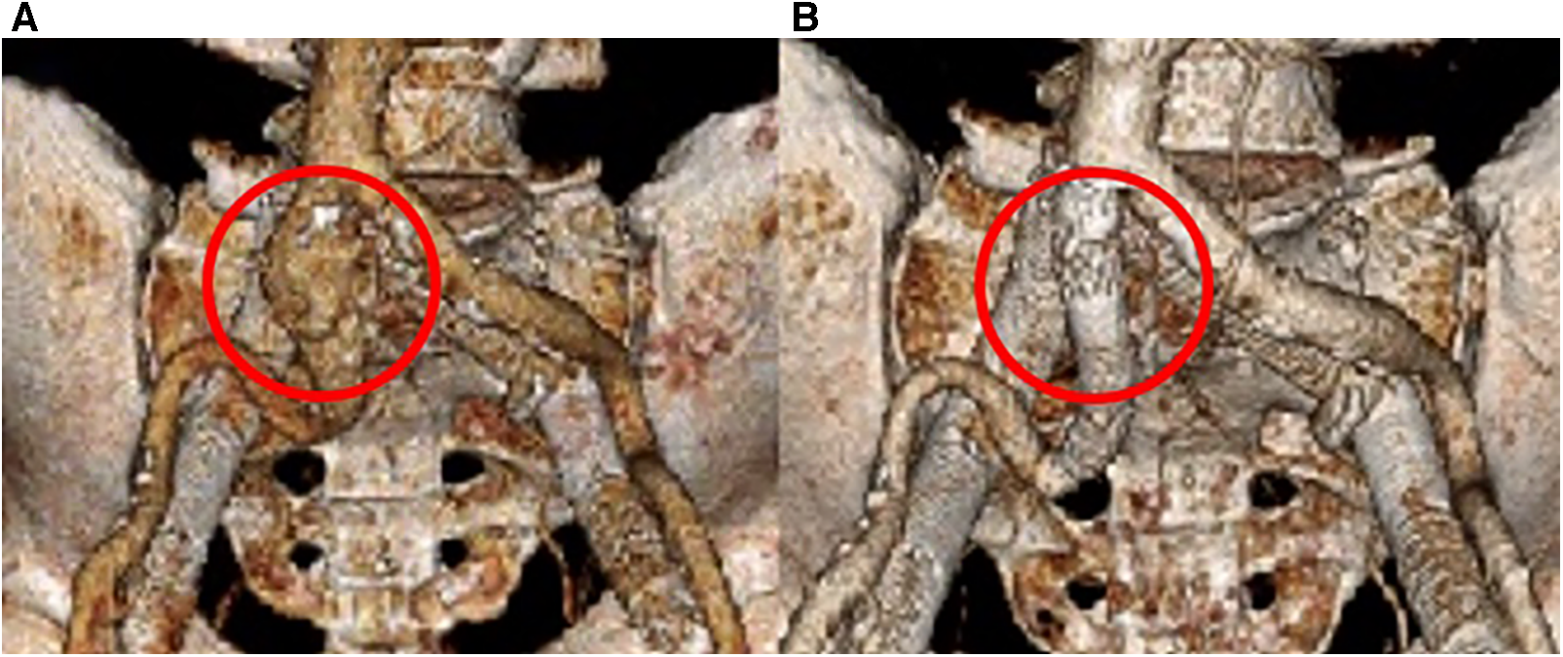
3D reconstruction of the arterial system. (**A**) Initial imaging revealing the right CIA aneurysm. (**B**) 18 months post-covered stenting shows a complete resolution of the aneurysm.

Follow-up imaging 6 months later demonstrated an increase in the size of the right CIA aneurysm (Figures 3B,C). Therefore, the patient was taken for a staged intervention of the aneurysm with the placement of covered iliac stents using a 13.5 mm Fluency self-expanding (Bard Peripheral Vascular, Tempe, AZ, USA) and 10 mm iCast stents (Atrium, Hudson, NH, USA) in overlapping fashion from the ostium of the right CIA to the bifurcation of the internal–external iliac arteries. These stents were chosen due to sizing availability at the time over the traditional EVAR limb. The Fluency stent was initially used as it would result in adequate coverage without an obvious endothelial leak. Once the covered stent was placed in the proximal CIA, we proceeded with placing a 1:1-sized iCAST stent to ensure appropriate coverage of the aneurysmal segment. Follow-up CTA demonstrated a regression and thrombosis of the aneurysm and patent, well-aligned venous stents, Figure 3D. The near resorption of the native aneurysm sac can be discerned from Figures 4A,B. Because of the placement of the stents and resumption of anticoagulation, the patient has had no further reoccurrences of DVTs or other related symptoms.

## Discussion

MTS is often underdiagnosed, acting insidiously until realized late into its pathologic findings. This, in part, is due to the various etiologies and resultant manifestations. Of these, numerous arterial causes exist, including the involvement of the native artery, arterial variants, iliac artery stent, or other endovascular stent graft, abdominal aortic, or iliac artery aneurysms—further denoted by right- or left-sided involvement, of which the right CIA is often the culprit. MTS secondary to CIA aneurysms is a relatively rare phenomenon and typically found only incidentally. Large aneurysms may predispose the individual to compression of surrounding structures, arterial thrombosis, or thromboembolism and rupture; however, venous disease is a rare manifestation of these aneurysms. Regardless, a few case reports exist in the literature describing the effects of right CIA aneurysm resulting in left CIV compression with associated DVTs (4).

Venous symptomatology is contingent upon the degree of obstruction, anatomical involvement, and absence of any collaterals to supplement efficient flow. If venous hemodynamics are acutely compromised, the affected limb may present with unilateral heaviness, swelling and venous claudication, and acute thrombosis, which may lead to phlegmasia cerulea dolens or phlegmasia alba dolens, requiring urgent intervention (2). Chronically, the patient may develop chronic venous insufficiency leading to DVTs, leg pigmentation, or venous ulcers if left untreated, post-thrombotic syndrome (1).

Although a vast majority of MTS presentations are clinically silent, thrombotic events may precipitate if transient risk factors or external stressors are present. Such factors include surgery, prolonged immobility, or a hypercoagulable state especially in the setting of underlying malignancy or with the initiation of oral contraceptives. In our case, the history of the hypercoagulable state and cessation of anticoagulation likely produced the perfect milieu for the compressive aneurysm to result in venous flow stagnation and thrombosis. Once identified, treatment is focused on improving venous outflow through the affected limb. Anticoagulation is initiated until the clot burden and extent of MTS can be assessed.

The CaVenT and a subgroup analysis of the ATTRACT trial demonstrated that catheter-directed thrombolysis, along with anticoagulation, has been deemed superior to just anticoagulation alone, particularly in extensive iliofemoral DVTs (1, 5). A 5-year follow-up of the CaVenT cohort continued to show increased clinical benefits in addressing disease severity (6). Current guidelines from the American Society of Hematology, American College of Chest Physicians, and American Heart Association do not support routine thrombolytic therapy and recommend anticoagulation alone in acute DVT, regardless of burden (7). However, in extensive and symptomatic thrombus refractory to or with contraindications to anticoagulation, mechanical thrombectomy can be used in adjunct to assist with reducing clot burden. In such cases of acute thrombosis, patients should undergo various optional percutaneous procedures (including thrombectomy or thrombolysis) followed by endovascular stent placement if indicated, as per the recommendations of the Society of Interventional Radiology and Society of Vascular Surgery (1). With regard to venous stenting, the ABRE study evaluated Abre venous stent use and demonstrated an overall 1-year primary patency of 88% and a 1-year primary assisted and secondary patency of 92.9% (8). Another study looked at the long-term clinical outcomes of Wallstent and found a primary patency of 89% and a primary assisted patency and secondary patency of 95% at 6 years (9).

As mentioned previously, numerous arterial etiologies have been attributed to MTS, including the CIA. Current recommendations for iliac artery aneurysms suggest treatment if symptomatic, for an aneurysm size of 3.5 cm with an increased risk for rupture, or for rapid aneurysmal expansion (10). Endovascular first-year and third-year primary graft patency rates are 97% (11). Although there are no benefits in terms of primary artery patency or mortality rate, the endovascular approach is preferred over the open vascular one due to decreased perioperative morbidity, operative time, and hospital stay (12). The other advantages include avoidance of operative complications such as sympathetic and parasympathetic nerve damage or disruption of lymphatics (13). As such, we opted for a completely percutaneous treatment modality over open surgical repair. Following endovascular repair, lifelong surveillance is required to assess stent patency or migration and monitor for endoleaks and aneurysmal diameter changes.

In our case, the initial aneurysmal size did not meet the repair threshold despite the symptomatic presentation. We addressed the acute concern that pertained to the venous system, by means of mechanical thrombectomy and venous stenting. Although the right CIA appeared to be the obvious culprit, a question arose as to how symptomatic the aneurysm was now that a venous stent was placed to ensure architectural integrity, with the possibility of averting arterial intervention. Given the size of the aneurysm, improvement post-venous intervention, and along with patient preference, a conservative approach was adopted with standard serial imaging done every 6 months. Unfortunately, the aneurysm demonstrated interval growth, necessitating arterial intervention with an arterial stent, the outcome of which was successful.

## Conclusions

The subtle presentation of MTS can make diagnosis challenging, especially in the presence of other more plausible explanations of DVTs. This is further made difficult by the numerous etiologies that can result in MTS. To our knowledge, this is a previously unreported presentation of bilateral MTS caused by a right-sided CIA aneurysm at the iliac venous confluence with resulting venous compression of both vessels managed in a complete endovascular fashion in terms of venous and arterial disease.

## Learning objective

1. Bilateral MTS is an uncommon etiology but should be kept in the differential of DVTs presenting bilaterally.
2. IVUs can clarify arterial and venous pathologies.
3. Endovascular management of venous compression syndrome may require arterial intervention.

## Data Availability

The original contributions presented in the study are included in the article/Supplementary Material, further inquiries can be directed to the corresponding author.
